# Spatiotemporal mapping of immune and stem cell dysregulation after volumetric muscle loss

**DOI:** 10.1172/jci.insight.162835

**Published:** 2023-04-10

**Authors:** Jacqueline A. Larouche, Emily C. Wallace, Bonnie D. Spence, Eric Buras, Carlos A. Aguilar

**Affiliations:** 1 Department of Biomedical Engineering,; 2 Biointerfaces Institute,; 3 Department of Molecular, Cellular and Developmental Biology,; 4 Division of Metabolism, Endocrinology and Diabetes, Department of Internal Medicine, and; 5 Program in Cellular and Molecular Biology, University of Michigan (UM), Ann Arbor, Michigan, USA.

**Keywords:** Muscle Biology, Stem cells, Adult stem cells, Fibrosis, Skeletal muscle

## Abstract

Volumetric muscle loss (VML) is an acute trauma that results in persistent inflammation, supplantation of muscle tissue with fibrotic scarring, and decreased muscle function. The cell types, nature of cellular communication, and tissue locations that drive the aberrant VML response have remained elusive. Herein, we used spatial transcriptomics on a mouse model of VML and observed that VML engenders a unique spatial profibrotic pattern driven by crosstalk between fibrotic and inflammatory macrophages and mesenchymal-derived cells. The dysregulated response impinged on muscle stem cell–mediated repair, and targeting this circuit resulted in increased regeneration and reductions in inflammation and fibrosis. Collectively, these results enhance our understanding of the cellular crosstalk that drives aberrant regeneration and provides further insight into possible avenues for fibrotic therapy exploration.

## Introduction

Severe extremity trauma resulting in volumetric muscle loss (VML) incites reductions in muscle function and quality of life (1, 2). The range and nature of VML injuries as well as alterations in structural and metabolic demands have obfuscated surgical repair schemas and regenerative therapies (3, 4). Currently, the cellular and molecular factors that drive the pathological VML response and prevent healing remain poorly understood. The frank loss of tissue after VML manifests in immune cell infiltration that lasts for days to months (5, 6) and is accompanied by fibrotic scarring (7, 8). This pathological outcome is in contrast to skeletal muscle injury that results in regeneration (9–11). Previous studies have demonstrated increases in infiltration and accrual of neutrophils (9), macrophages (Møs) (6, 8, 12, 13), and Th2 (14) cells in VML injury, and the adverse effects of the sustained inflammation from these cells range from exacerbated tissue damage to prevention of muscle stem cell–mediated (MuSC-mediated) repair (9). As such, quantitative mapping of immune and stem/progenitor cell dysfunction after VML and how intercellular communication is spatially modified to inhibit regeneration is needed to maximize tissue repair schemas.

Spatial transcriptomics (spGEX) is a technology that generates unbiased RNA-Seq data sets in a spatially registered manner via capture of transcripts on barcoded beads that are decorated with DNA capture probes and tethered to specific locations on a glass slide (15). This technique is amenable to integrate with histological staining and imaging, rendering coupled insights into pathological mechanisms of expression changes with morphological context. spGEX has revealed critical aspects of how injury-responsive cells are recruited to the wound (16), alter their state, and signal with other cell types during tissue repair. However, exploration of spGEX in the pathological microenvironment of VML injured muscle to elucidate regional variations of immune cell and progenitor functions after VML has not been performed.

Herein, we profiled murine VML defects over a time course using spatial spGEX integrated with single-cell RNA-Seq (scRNA-Seq) data sets to understand the spatial context underlying the development and progression of muscle fibrosis. We observed and validated a spatial patterning of scar-associated Møs (SAMs) colocalized with mesenchymal-derived cells (MDCs) within VML defects and negligible infiltration of myogenic cells at 7 days postinjury (dpi). Cell communication analysis revealed Møs as key drivers of profibrotic signaling, with a unique subset of Trem2^+^ Møs that have been observed in other fibrotic contexts. At 14 dpi, spGEX showed that inflammation had largely resolved while fibrosis ensued, and there was an accumulation of MDCs in the VML defect region. Finally, inhibition of profibrotic signaling using a small molecule inhibitor of TGF-β receptor 2 resulted in increased infiltration of MuSCs into the VML defect, along with reduced inflammatory and fibrotic signaling transcripts. Together, this work provides a resource for further understanding cell-to-cell communication networks that contribute to fibrotic degeneration in a spatial context.

## Results

### spGEX of murine VML injury reveals cellular and molecular pathology.

**Figure 1:**
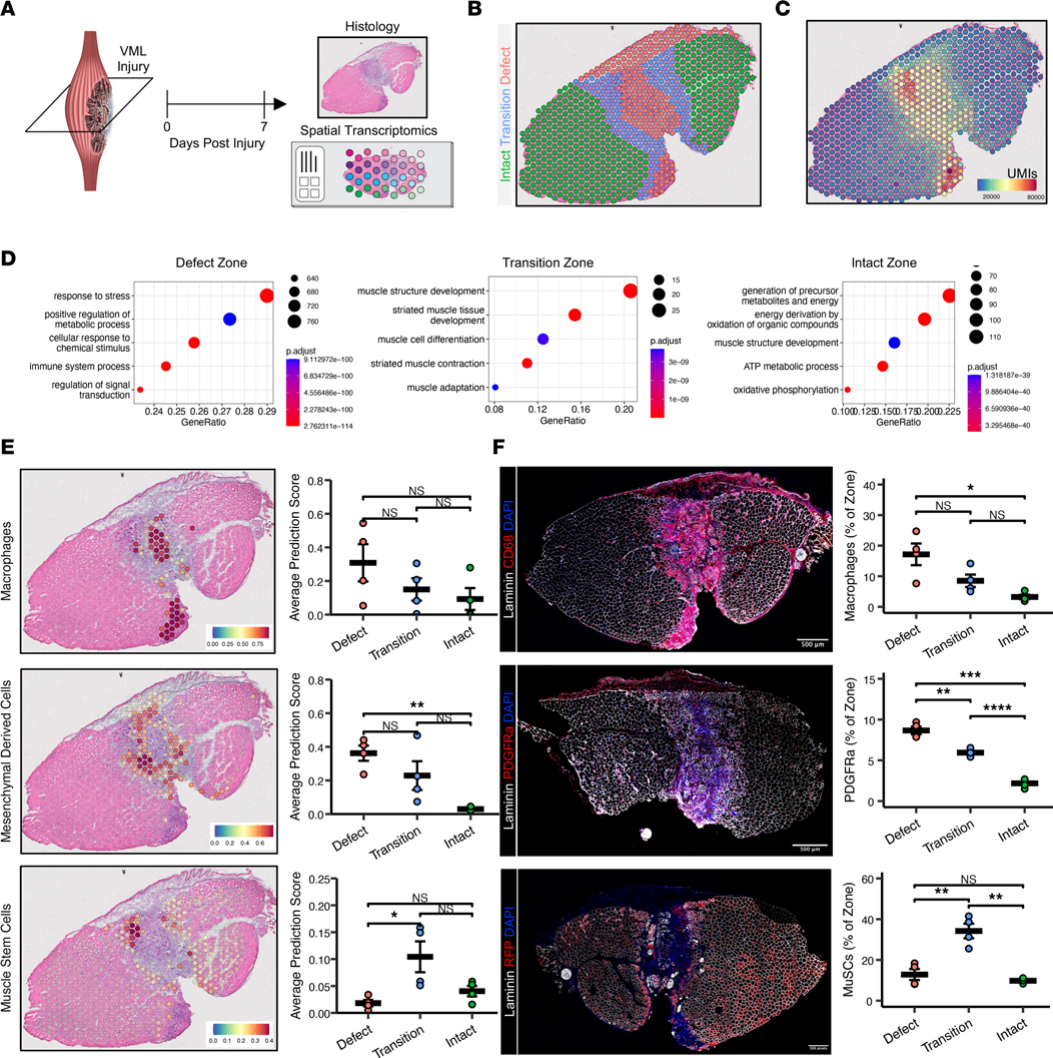
Spatial transcriptomic profiling 7 days after VML reveals profibrotic spatial patterning in the injured site. (**A**) Experiment schematic whereby spatial transcriptomics was performed on VML-injured tibialis anterior muscles at 7 days after injury. (**B**) Representative image showing tissue annotation into 3 zones — a defect zone, a zone of intact muscle, and a transition zone between them. (**C**) Representative distribution of unique molecular identifiers shows higher read counts at the location of the defect. (**D**) GO Term analysis showing upregulated terms within each zone compared with the other 2 zones. Differentially expressed genes were calculated using Wilcoxon Rank Sum Test with post hoc analysis. Log_2_ fold change > 0.25 and adjusted *P* < 0.05 was considered significant. (**E**) Integration of spatial transcriptomics data sets with matched, cell type–annotated scRNA-Seq data sets using Seurat label transfer. (Left) Representative spatial overlays. (Right) Quantification. **P < 0.01, *P < 0.05, by 1-way ANOVA with Tukey’s post hoc analysis. *n* = 4 muscles from 2 male and 2 female mice. Color bars indicate prediction scores. (**F**) Immunohistological stains confirm the spGEX-predictions of cell localization within the different zones. (Left) Representative images. Scale bars: 500 μm. (Right) Quantifications. **P < 0.01, *P < 0.05, by 1-way ANOVA with Tukey’s post hoc analysis. *n* = 3–4 muscles from 2 male and 2 female mice. ***P < 0.001, ****P < 0.0001. Magnification, 20×.

To understand the fibrotic response that develops after VML, we administered 2 mm full-thickness punch biopsies to the tibialis anterior (TA) muscles of young adult mice. Consistent with previous results (9, 17, 18), we observed increased and persistent interstitial fibrosis as a result of VML injuries by staining with Picrosirius red at 0, 7, 14, and 28 dpi (Supplemental Figure 1; supplemental material available online with this article; https://doi.org/10.1172/jci.insight.162835DS1) (Supplemental Table 3). To decipher the mechanisms that confer the fibrotic behavior, we extracted cryosections of VML-injured murine tissues from 7 dpi, stained for H&E, and annotated the tissue into zones of (a) complete muscle loss (defect zone), (b) remaining in-tact muscle (intact zone), and (c) a transition zone that partitions the lost muscle from the remaining musculature (Figure 1, A and B). The defect zone was characterized by an abundance of mononucleated cells, consistent with previous observations of inflammation (9), while the intact zone contained muscle fibers with peripherally located nuclei. The transition zone was characterized by both mononucleated cells and myofibers with centrally located nuclei, indicating active regeneration or degeneration. To glean insights into the complex signaling milieu occurring within these zones, we generated replicate spatial transcriptomic maps on 4 VML injured tissues at 7 dpi using the 10X Genomics Visium platform. We generated 247,976,765 sequencing reads, mapped the demultiplexed reads to their corresponding spatial location, and observed 2,939 location-specific barcodes with a median of about 13,000 unique molecular identifiers (UMIs) and 3,500 unique genes per spot (Supplemental Table 1). Reads derived from the defect zone displayed more UMIs than the other 2 zones, consistent with the increased number of cells in these locations (Figure 1C). We performed gene set enrichment analysis on each zone and observed enrichments in gene sets associated with the immune system, stress, and defense in the defect zone; muscle structure development in the transition zone; and various metabolism gene sets in the intact muscle zone (Figure 1D). Toward this extent, genes associated with inflammation (*Ctss*, *S100a8*, *S100a9*) and collagen deposition (*Col1a1*, *Col1a2*) were enriched in the defect zone, whereas developmental myogenic genes (*Tnnt1*, *Tnnt2*, *Myh3*, *Myh8*) were upregulated in the transition zone, and metabolism genes (*Cox6a2*, *Cox8b*) and genes associated with mature muscle fibers (*Myh4*) were upregulated in the intact zone (Supplemental Figure 2A). These results demonstrate that spatial gene expression (spGEX) programs following VML injury are consistent with tissue morphology, indicating an area of inflammation, a region of regeneration, and a region that is morphologically intact but exhibits altered metabolism in response to the defect.

A current limitation of spGEX is the low resolution of spatially barcoded spots, which contain reads from up to 10 proximal cells (19). To probe cell localization within our spatial data sets, we used Seurat to integrate our spGEX data with scRNA-Seq data sets of VML defects isolated at 7 dpi that we previously generated (9, 20). This analysis revealed regional localization of cell types consistent with GO Term enrichment analysis and differential gene expression analysis (Figure 1E; *n* = 4 tissues from 4 mice, 2-sample, 2-sided *t* test). For example, the defect zone was predicted to be predominantly occupied by Møs and MDCs, consistent with the localization of genes such as *Cd68*, *Adgre1*, *Aspn*, and *Col1a1* (Supplemental Figure 2, B and C). The transition zone was primarily occupied by MuSCs and their progeny, expressing transcripts associated with myogenesis including *Myog* and *Myod1* (Figure 1E and Supplemental Figure 2, B and C). We did not detect expression of MuSC marker genes in the defect zone, and this may indicate cellular signaling that inhibits migration or regenerative actions of these cells. To confirm these predictions, we performed immunohistological staining for CD68^+^ Møs and observed nearly identical patterns as our spGEX analysis (Figure 1F; *n* = 4 tissues from 4 mice, 1-way ANOVA with Benjamini-Hochberg [BH] post hoc analysis), whereby the defect zone exhibited enrichments in CD68^+^ Møs. To validate MDCs, we utilized a fluorescent reporter mouse model for PDGFRa^+^ cells (PDGFRa^EGFP^), which express an H2B-eGFP fusion gene and display green nuclei. PDGFRa^EGFP^ mice were administered VML defects as above, cross-sections of the VML injured tissues were isolated at 7 dpi, and immunostaining for eGFP^+^/PDGFRa^+^ mesenchymal cells was performed. Similar to our observations with Møs, we detected localized enrichment of eGFP^+^/PDGFRa^+^ cells in the defect and transition zones (Figure 1F; *n* = 5 tissues from 5 mice, 1-way ANOVA with BH post hoc analysis). To verify positioning of MuSCs and their progeny after VML, we employed a lineage-tracing system for Pax7^+^ cells (21) (Pax7^CreERT2^-Rosa26^TdTomato^) and generated VML defects as above. Cross-sectioning and immunostaining for TdTomato at 7 dpi revealed highly concordant results with our spGEX analysis, whereby MuSCs and their progeny were enriched in the transition zone and were almost entirely absent from the defect region (Figure 1F; *n* = 4 tissues from 4 mice, 1-way ANOVA with BH post hoc analysis). These results suggest that MuSCs are either inhibited from entering the defect zone or otherwise are unable to remain.

### Intercellular signaling within the VML injury site is profibrotic and driven by dysfunctional Møs.

**Figure 2:**
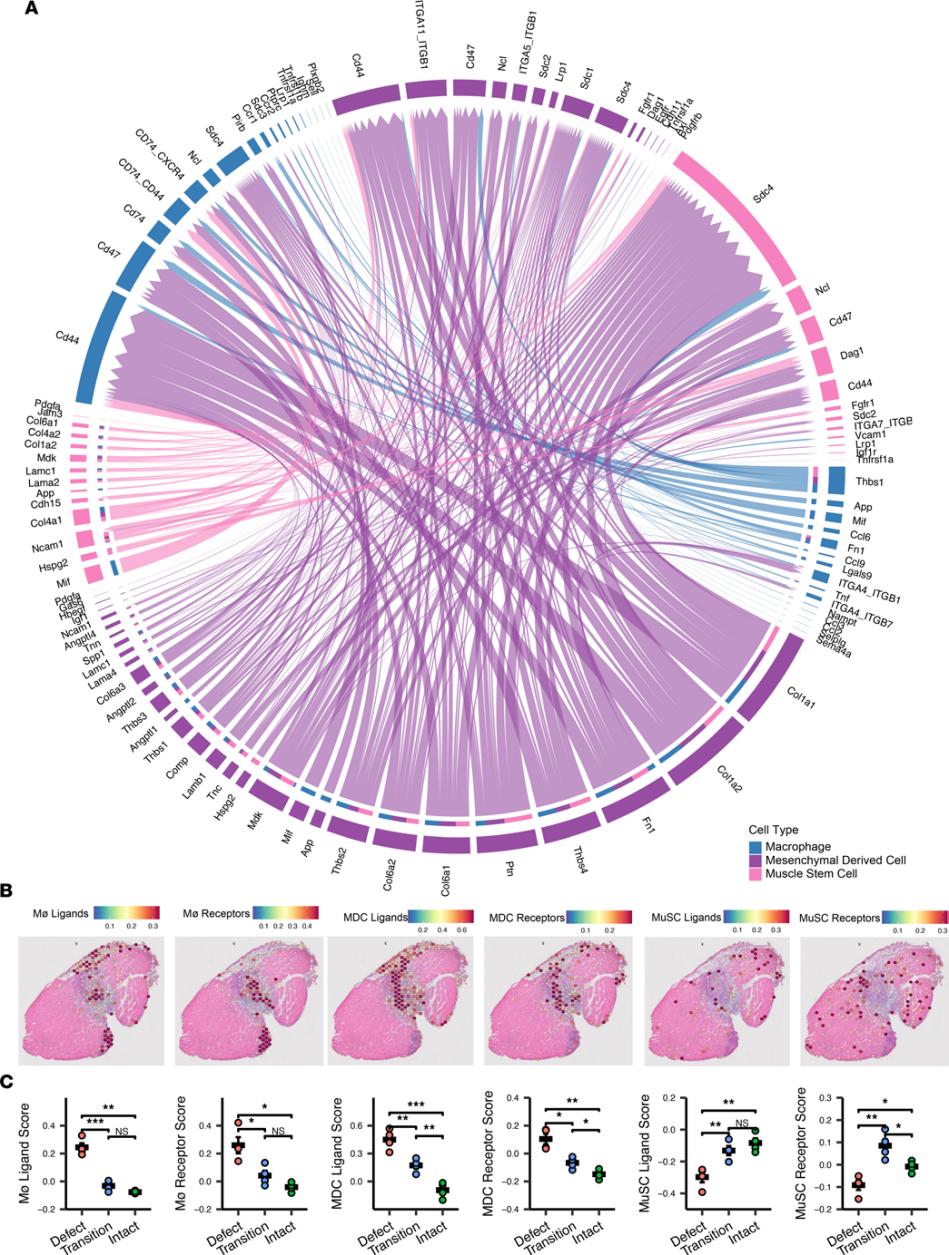
Signaling pathways between macrophages, mesenchymal-derived cells, and muscle stem cells after VML are predominantly profibrotic at 7 dpi. (**A**) Chord diagram displaying all significant interactions between macrophages, mesenchymal-derived cells, and muscle stem cells determined using CellChat on the scRNA-Seq reference data set. Interactions with *P* < 0.05 based on CellChat’s permutation test were considered significant. (**B**) Representative gene module overlays for the ligands and receptors predicted to be involved in significant cell-to-cell interactions show spatial proximity in the defect and transition zones. (**C**) Average ligand and receptor module scores across zones demonstrates higher scores for Mø and MDC signaling molecules in the defect zone, increased mesenchymal-derived signaling molecules in the transition zone compared with the intact zone, and higher MuSC module scores in the transition and intact zones. ***P < 0.001, **P < 0.01, *P < 0.05, by 1-way ANOVA with Tukey’s post hoc analysis. *n* = 4 tissues from 2 male and 2 female mice. Magnification, 20×.

To gain deeper insights into intercellular signaling that regulates the regenerative response, we assessed ligand-receptor interactions between predicted cell types in the defect and transition zones. Specifically, we subset the Møs, MDCs, and MuSCs from the 7 dpi scRNA-Seq reference data set and performed CellChat (22) interaction analysis. CellChat inputs the results of differential expression testing between cell types into a mass action model to quantify communication probability and infer statistically and biologically significant cell-to-cell communication pathways and signaling roles between different cell types. Moreover, the curated database input into the communication model accounts for the impacts of multimeric ligand-receptor complexes, soluble agonists and antagonists, and stimulatory and inhibitory membrane-bound coreceptors on signaling pathway activation or deactivation. We observed substantial crosstalk between the 3 cell types, and we found that the majority of the signaling ligands were expressed by the MDCs (Figure 2A). Moreover, most of these signaling ligands are profibrotic, including various thrombospondins (*Thbs1*, *Thbs2*), collagens, and extracellular matrix proteins (*Col1a1*, *Col1a2*, *Col6a1*, *Col6a2*, *Comp*, *Fn1*). We also observed various inflammatory ligands and receptors, predominantly expressed among the Møs (*Ccr1*, *Ccr2*, *Ccl2*, *Ccl3*, *Ccl6*, *Ccl9*, *Tnf*, *Tnfrsf1a*, *Tnfrsf1b*). To localize these transcripts within the tissues, we calculated module scores for the expression of the CellChat-predicted ligands and receptors and overlaid them onto the H&E images (Figure 2B). This revealed colocalization of the mRNAs for cell type–specific ligands and receptors in the same regions, confirming that the spatial proximity and concentration of these genes in the defect and transition zones supports a profibrotic communication network following VML (Figure 2C; *n* = 4 tissues from 4 mice, 1-way ANOVA with BH post hoc analysis).

**Figure 3:**
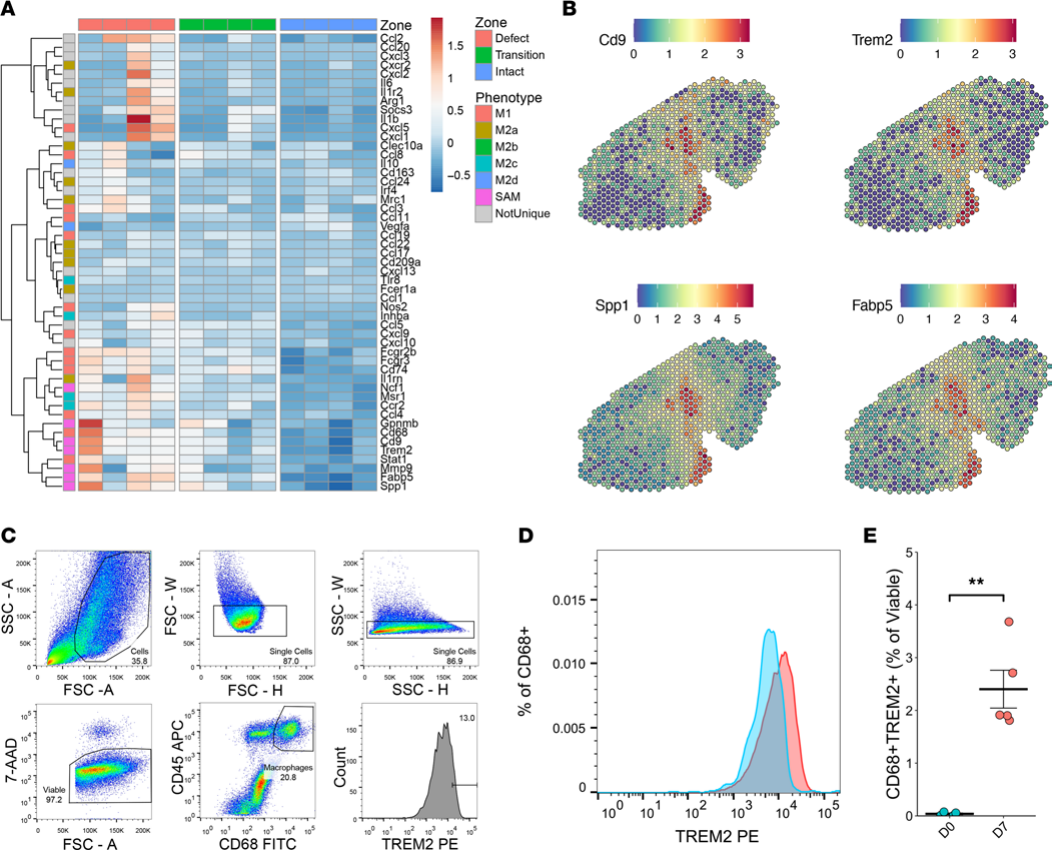
Macrophage phenotype at 7 dpi aligns predominantly with scar-associated macrophages. (**A**) Heatmap of the expression of genes associated with different macrophage polarizations across the defect, transition, and intact muscle zone. Scale indicates *Z* score of average gene expression for each replicate in each zone. *n* = 4 tissues from 2 male and 2 female mice. (**B**) Representative overlays of marker genes for SAMs support localization primarily in the defect. Color bars show SCT-transformed expression. (**C**) Flow cytometry gating schematic for detecting CD68^+^TREM2^+^ macrophages. (**D**) Representative histogram of TREM2 expression among CD45^+^CD68^+^ cells for uninjured versus 7 dpi VML-injured tissue. (**E**) Quantification of CD68^+^TREM2^+^ macrophages as a fraction of viable cells. **P < 0.01 by 2 sided, 2-sample *t* test. *n* = 4–5 muscles from 2–3 mice.

Since Møs and MDCs were colocalized in the VML defect and promoted a profibrotic signaling environment, we analyzed the Mø population in further detail. Møs play a critical role in coordinating healing versus fibrotic outcomes in skeletal muscle after injury through transitions from a proinflammatory state (M1) into various proregenerative or profibrotic states (M2) typically denoted as M2a, M2b, M2c, and M2d subsets. More recently, a transcriptionally distinct Mø phenotype (SAM) with characteristics of both the M1 (proinflammatory) and M2 (prorepair) phenotype criteria has been associated with fibrosis of various tissues, including cardiac muscle (23, 24), liver (25), and lung (26). To understand the polarization of Møs within VML defects at 7 dpi, we evaluated the expression of previously reported marker genes of Mø phenotype (27) in addition to the expression of SAM markers, such as *Trem2* and *Spp1*. We observed considerable upregulation of SAM-specific transcripts in the defect zone, with few Mø polarization marker genes expressed in the transition or intact zones, consistent with cell type annotation results (Figure 3, A and B). To confirm that SAMs are present within VML-injured tissues at 7 dpi, we performed flow cytometry for CD45^+^CD68^+^TREM2^+^ cells (Figure 3C). Consistent with our spGEX analysis, TREM2^+^ Møs were detected in VML-injured tissues compared with uninjured murine TAs (Figure 3, D and E; *n* = 4–5 muscles from 2–3 mice, unpaired). Together, these results suggest that SAM and MDC occupancy of the defect region creates an inflammatory and profibrotic milieu that potentially inhibits MuSC-mediated regeneration.

### Spatiotemporal progression of VML injury shows reductions in inflammation and persistent interstitial fibrotic remodeling.

**Figure 4:**
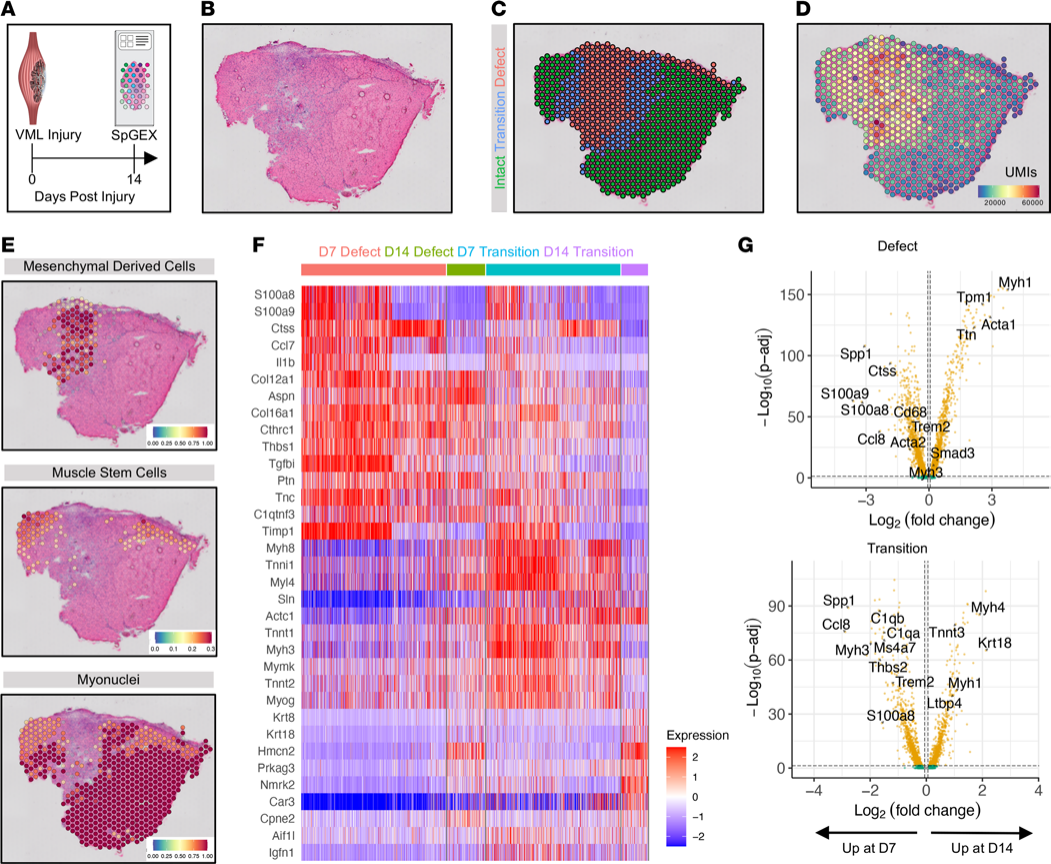
Spatial transcriptomic profiling 14 days after murine VML reveals reduced inflammation and persistent fibrotic remodeling. (**A**) Schematic of experiment design whereby mice were administered VML injuries to the TA muscles, and the injured TAs were collected at 14 dpi for spatial transcriptomics analysis. (**B**) Representative image of H&E-stained section of VML defect at 14 dpi. (**C**) Representative tissue annotation into the 3 zones — a defect zone, a zone of intact muscle, and a transition zone between them. (**D**) Distribution of unique molecular identifiers shows higher read counts at the location of the defect. (**E**) Integration of spatial transcriptomics data sets with matched, cell type–annotated scRNA-Seq data sets using Seurat label transfer predicts the defect zone being predominantly inhabited by profibrotic mesenchymal-derived cells. Muscle stem cells are still largely absent from the defect zone but localize in the intact muscle zone. Scales indicate prediction scores. Representative of 2 replicates from 1 male and 1 female. (**F**) Heatmap of differentially expressed genes by zone and time point highlights loss of inflammatory transcripts within the defect zone by 14 dpi but continued expression of profibrotic genes. The transition zones at 7 dpi and 14 dpi were transcriptionally distinct, with more active regeneration occurring at 7 dpi. Color bar shows scaled expression (*Z* scores). (**G**) Volcano plots showing substantial differential gene expression across time points in both the defect (top) and transition (bottom) zones. Inflammatory genes are downregulated by 14 dpi in both zones, while myogenic terms are upregulated. Yellow indicates log_2_ fold change greater than 0.0585 and adjusted *P* < 0.05, which was considered significant. Green indicates log_2_ fold change greater than 0.0585 and *P* > 0.05. Magnification, 20×.

To understand the fibrotic progression of VML and whether defect-localized signaling changes with time, we generated spGEX maps of murine VML defects at 14 dpi (Figure 4A). We generated 94,467,583 sequencing reads, which mapped to 1,277 location-specific barcodes across 2 tissues, with a median of about 15,000 UMIs and about 4,000 unique genes per spot (Supplemental Table 2). The defect and transition zones were annotated based on tissue morphology as described above (Figure 4, B and C) and displayed higher numbers of reads per spot compared with the intact zone (Figure 4D). This was again consistent with increased density of mononucleated cells within the defect and transition regions. The spGEX spots were annotated into various cell types by integrating matched 14 dpi VML scRNA-Seq data sets (9, 20). The defect zone at 14 dpi was primarily annotated as MDCs, with MuSCs and myonuclei occupying the transition and intact zones (Figure 4E and Supplemental Figure 3A). Mø marker genes — including those specific to SAMs (Supplemental Figure 3C) — were also strongly detected within the defect zone (Supplemental Figure 3B). These results suggest the signaling milieu within the VML defect remains primarily profibrotic and is consistent with histological observations showing exacerbated collagen deposition at 14 dpi (Supplemental Figure 1).

To glean variations between 7 and 14 dpi, we integrated the data sets from both time points and observed clustering by time points and tissue annotation/location (Supplemental Figure 3D). To understand transcriptional differences across zones and time points, differential expression testing was performed to identify the top upregulated genes for each zone and time point (7 dpi defect, 14 dpi defect, 7 dpi transition, 14 dpi transition), excluding the regions of intact muscle. This revealed a loss of inflammation-associated transcripts (*S100a8*, *S100a9*, *Ctss*, *Ccl7*, *Il1b*) within the defect zone at 14 dpi but revealed persistent upregulated expression of profibrotic transcripts (*Aspn*, *Thbs1*, *Col12a1*, *Col16a1*) (Figure 4F). The transition zones at 7 and 14 dpi were transcriptionally more distinct, with genes associated with active regeneration upregulated (*Myog*, *Mymk*) at 7 dpi (Figure 4F). To visualize differences across time, we performed differential gene expression testing for each zone (Figure 4G). We detected significant downregulation of genes associated with inflammation and SAMs, such as *Ctss*, *Cd68*, *Ccl8*, *S100a8*, *S100a9*, *Trem2*, and *Spp1*, in the defect zone at 14 dpi. We also observed reduced expression of complement genes and genes associated with inflammation, including *Ms4a7*, *S100a8*, and *Ccl8*, in the transition zone at 14 dpi. Compared with 7 dpi, the defect and transition zones at 14 dpi also upregulated transcripts associated with mature muscle fibers, such as *Myh1*, *Myh4*, *Tpm1*, *Tnnt3*, *Krt18*, *Ttn*, and *Acta1*. GO Term analysis on differentially expressed genes in the defect and transition zones across time points were consistent with elevated inflammatory and stress terms at 7 dpi and increased muscle development and regeneration terms at 14 dpi (Supplemental Figure 3E). The persistence of fibrotic gene expression and increase in myogenic terms aligns with tissue morphology at 14 dpi, where interstitial fibrosis is abundant, while some myofibers have begun to repopulate around the defect (Figure 4B and Supplemental Figure 1). Together, these results indicate that the substantial resolution of inflammation by 14 dpi in the TA VML model facilitates some MuSC-mediated regeneration but that the fibrotic program that yields long-term functional deficits (9, 18) has already been established.

### Interruptions to TGF-β modulates spatial crosstalk that contributes to fibrosis.

**Figure 5:**
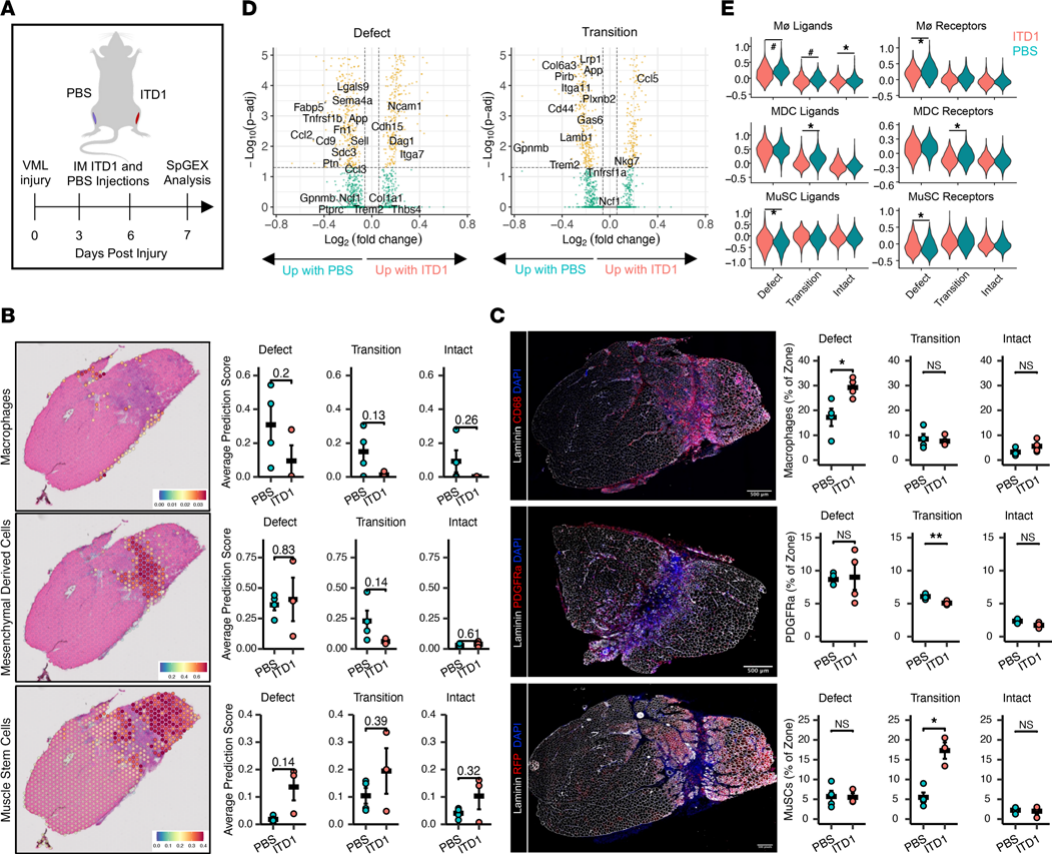
TGF-β signaling inhibition reduces inflammation and enhances myogenesis within the VML defect. (**A**) Experiment schematic whereby a cohort of mice received bilateral VML defects to the tibialis anterior muscles followed by intramuscular injection of vehicle (PBS) or TGFBR2-inhibitor ITD1. Spatial transcriptomics analysis was performed at 7 dpi. (**B**) Integration of spatial transcriptomics data sets with matched, cell type–annotated scRNA-Seq data sets using Seurat label transfer. Plots are annotated with *P* values. *n* = 3–4 tissues from 3 male and 3 female mice. Statistics performed using 2-sided, 2-sample *t* test. Color bars indicate Seurat prediction scores. *P* < 0.05 were considered significant. (**C**) (Left) Representative immunohistological stains of macrophages (CD68), PDGFRa^+^ mesenchymal cells, and RFP^+^ MuSCs and their progeny from tissues treated with ITD1. Scale bars: 500 μm. (Right) Quantifications of immunohistological images. *P < 0.05, **P < 0.01. *n* = 3–4 tissues from 3 male and 3 female mice. Statistics performed using 2-sided, 2-sample *t* test. (**D**) Volcano plots showing differential gene expression as a result of ITD1 treatment in the defect (left) and transition zones (right). Differential expression was calculated using MAST, and genes with a adjusted *P* < 0.05 were considered significant (yellow). Green indicates log_2_ fold change greater than 0.0585 and *P* > 0.05. (**E**) Violin plots of gene module scores for the macrophage, mesenchymal-derived cell predicted to be involved in active signaling pathways after VML. *P < 0.05, ^#^*P* = 0.06, by 2-sample, 2-sided *t* test comparing average module scores for each tissue in each zone across treatments. *n* = 3–4 tissues per group from 2 male and 2 female mice. Magnification, 20×.

Based on the observed activation of fibrotic signaling between Møs, MDCs, and MuSCs after VML and prior observations of improved functional recovery, enhanced myogenesis, and reduced fibrosis after TGF-β receptor 2 (TGFBR2) inhibition in mice (9, 28), we sought to understand the spatial and signaling implications of pharmacologically inhibiting TGFBR2. A cohort of mice received bilateral VML defects to the TA followed by treatment with ITD1, which is a selective molecule that promotes degradation of TGFBR2 in 1 limb and vehicle in the contralateral limb (Figure 5A). At 7 dpi, we stained sections with H&E to observe and annotate tissue morphology into the 3 zones and generated matched spGEX data sets (Supplemental Figure 4, A–C). Consistent with improvements in function and reduced fibrosis, gene set enrichment analysis comparing the defect zone in treated and untreated TAs showed upregulation of terms associated with myogenesis and muscle repair (Supplemental Figure 4D). Integration with scRNA-Seq data sets to localize cell populations across the tissues predicted reductions in Møs throughout the tissue, reductions in MDCs in the transition zone, and the presence of myogenic cells in the defect zone (Figure 5B; *n* = 4 tissues from 4 mice, 2-sample, 2-sided *t* test). Immunofluorescent staining of serial tissue sections confirmed the spGEX predictions of reduced MDCs and increased myogenic cells following ITD1 treatment, though CD68^+^ Møs were increased in the defect zone (Figure 5C; *n* = 3–4 tissues from 3–4 mice, 2-sample, 2-sided *t* test). This result may be driven by transcriptional differences in the Mø populations or by increased abundance of cell types other than Møs, including increased myogenic cells, as a result of ITD1 treatment. Thus, to understand whether Mø phenotype was altered based on treatment, we compared the expression of Mø polarization markers between ITD1- and vehicle-treated tissues (Supplemental Figure 4E). This analysis suggested that SAMs remain the dominant Mø phenotype, though the module score derived from SAM marker genes was lower following ITD1 treatment (Supplemental Figure 4F). M1 transcripts were downregulated in all ITD1-treated tissues, unlike post–vehicle treatment (Supplemental Figure 4E), suggesting that the treatment has an antiinflammatory impact on the Møs in addition to being antifibrotic. This suggests that inhibiting TGF-β signaling via TGFBR2 reduces the inflammatory and profibrotic cellular profile within the defect zone that is preventing a MuSC-mediated regenerative response.

To further understand the implications of ITD1 treatment on regeneration after VML, we performed a MAST (29) differential gene expression analysis on the defect and transition zones (Figure 5D). In line with increased myogenic cells populating the defect, we observed increased *Cdh15* and *Ncam1* expression along with reduced inflammatory signaling genes (*Ptprc*, *Ccl2*, *Ccl3*, *Ccl9*, *Gas6*, *Tnfrsf1b*, *Lgasl9*, *Sema4a*). Within the transition zone, ITD1 treatment reduced expression of several of the profibrotic ligands and receptors predicted by CellChat (*Cd44*, *Tnfrsf1a*, *Gas6*). Interestingly, among the upregulated genes were *Nkg7* and *Ccl5*, which are associated with NK cells (30) and cytolytic NK cell signaling after VML (9). Overall, ITD1 treatment reduced many of the profibrotic signaling ligands and receptors predicted to be activated after VML by CellChat (Figure 5E); it reduced Mø ligands in all zones, reduced Mø receptors in the intact zone, and reduced MDC ligands and receptors in the transition zone. In line with improved regeneration and an enhanced myogenic response, MuSC ligands and receptors were increased in the defect zone after treatment (*n* = 4 tissues from 4 mice, 2-sample, 2-sided *t* test). Collectively, our results show that blocking TGF-β signaling creates a biochemical environment in the defect zone more amenable to the MuSC regenerative response, acting on both the immune and mesenchymal cell accumulation and localization, and favorably altering signaling networks between Møs, MDCs, and MuSCs.

## Discussion

VML has consistently demonstrated a failure of regeneration (17), fibrotic scarring (5, 8), and reductions in muscle function (2), but the cellular and molecular mechanisms that confer this behavior (7) and their spatial context remain poorly understood. As a result, many therapeutics for VML have displayed limited improvements in muscle regeneration and functional output (3, 4). Herein, we address this need using spGEX analysis of an injury time course in a murine VML model. We detected spatial transcriptional patterns that suggest that a driving role of Mø-MDC signaling contributes to fibrotic progression, impinging on MuSC-mediated regeneration. Targeting the cellular crosstalk between Møs and MDCs through TGF-β inhibition facilitated infiltration of MuSCs into the defect and dampened fibrosis.

The microenvironment is known to impact cellular behavior, and understanding cellular neighborhoods and which cell types colocalize in morphological regions that become dysregulated after VML is critical to understanding signaling mechanisms and informing therapeutic development (31). Herein, we identified a spatial patterning within VML defects whereby Møs and MDCs heavily populate the region, while myogenic cells inhabit the region between the defect and remaining intact muscle. The absence of MuSCs in the defect region at 7 dpi may be a result of an inability to migrate into or reside within the defect. We speculate that this behavior may be mediated by an inadequate biophysical microenvironment (32) (increased tension of the matrix or lack of binding sites) and/or an inhibitive biochemical microenvironment (33) driven by Mø-MDC signaling (34). A highly similar program has been observed in chronic muscle fibrosis, whereby inflammatory Møs secrete latent TGF-β1 that is activated by Fibro-Adipogenic Progenitors (FAPs)-secreted factors, which in turn induce FAP differentiation toward myofibroblasts (35). The mechanically stiffened extracellular matrix (36) and lack of available guidance cues in the VML defect (37, 38) may contribute to the lack of MuSC infiltration. Our results of colocalization of Møs and MDCs within the defect, the predominantly profibrotic ligand-receptor pairs, and the reduction of FAPs and MDCs with TGF-β inhibition suggests that similar mechanisms occur after VML (39). Furthermore, we observed increases in expression of NK cell–related transcripts with ITD1 treatment. Prior work from our group has shown that NK cell infiltration into VML injuries reduces neutrophil abundance and that sustained exposure to the neutrophil secretome impairs myogenesis in vitro (9). Thus, enhanced NK cell activity and restrained inflammation from neutrophils as a result of TGFBR2 inhibition could be a mechanism through which this intervention contributes to a more favorable biochemical environment for muscle regeneration. Inhibiting TGF-β signaling is likely also directly impacting MuSC function to confer improvements in regeneration. Further delineation of the mechanisms through which TGF-β signaling inhibits amelioration of fibrotic signaling by other cell types promotes regeneration remains an open question.

The role of Mø phenotype and dysfunction that develops after VML has not been sufficiently studied; therefore, there is a void in our understanding of how Møs contribute to fibrosis. Recent in vivo analyses and scRNA-Seq studies have elucidated a subset of Møs that display strong profibrotic activity (40). This profibrotic Mø subset, or SAM, displays unique surface markers (41) (Trem2, Spp1), has been colocalized with myofibroblasts (26), and has been shown to communicate through TGF-β1 (42). Additionally, SAMs have been detected in a variety of fibrotic and metabolic disease contexts such as cirrhosis (43), idiopathic pulmonary fibrosis (44), atherosclerosis (45) and Alzheimer’s disease (46), obesity (47, 48), and other pathologies. Our observation of SAMs within VML defects and colocalization with MDCs suggest that these cells may shape fibrosis during the VML response. Uniquely, SAMs have been found to be activated by extended association with neutrophils (26), which is consistent with our previous findings (9) of persistent neutrophil infiltration. Given that SAMs and neutrophils are lipid-sensitive cells (9, 18, 47), additional exploration of the temporal recruitment and programming of Trem2^+^ Møs (49) by extracellular lipids after VML is warranted.

VML continues to remain a significant clinical need, and our results enhance understanding of the pathological drivers of this trauma. These data sets may yield enhancements in existing regenerative therapies and may bring about useful aids for quantifying therapeutic effects for VML.

## Methods

### Animals

C57BL/6 WT (14 female and 14 male) and PDGFRa^EGFP^ female and male (3 females and 3 males) mice were obtained from The Jackson Laboratory or a breeding colony at the UM. Pax7Cre^ER/+^-Rosa26^dTomato/+^ mice (3 females and 3 males) were obtained from a breeding colony at UM and administered 5 daily 100 μL i.p. injections of 20 mg/mL tamoxifen in corn oil. All mice were fed normal chow ad libitum and housed on a 12:12-hour light/dark cycle under UM veterinary staff supervision. Equal numbers of male and female animals were used for each experiment. Chemical, peptide, and recombinant protein manufacturer information can be found in Supplemental Table 3.

### Injury model

Mice were anesthetized with 1.5% isoflurane and administered 0.1 mg/kg buprenorphine in 100 μL saline via i.p. injection. Puralube ointment was applied to both eyes. Hair was removed from the hindlimbs using Nair hair-removal cream. The surgical area was sterilized 3 times with Providone Iodine followed by 70% ethanol. A 0.5 cm incision was made in the skin on the anterior side of each TA muscle, followed by the removal of a 2 mm full-thickness muscle section from the middle of the TA muscle. The skin was sutured closed using 6-0 proline sutures, which were removed 7 days after surgery. At experiment endpoints, either uninjured, 7, 14, or 21 dpi, mice were humanely euthanized in accordance with the NIH and University Committee by asphyxiation followed by cervical dislocation, bilateral pneumothorax, and excision of the heart.

### Histology

#### Tissue sectioning.

TA muscles were extracted by blunt dissection using sterile surgical tools within 30 minutes of euthanasia. Tissues/biopsies were immediately simultaneously flash frozen and embedded in optical cutting temperature (OCT) compound according to 10X Genomics Demonstrated Protocol CG000240 Revision D. Frozen tissues were stored at –80°C. Serial cross sections were cut using a cryotome at –20°C at the midpoint of the injury and collected on positively charged glass slides (Thermo Fisher Scientific, 12-550-15). Duplicate cross sections from each tissue were placed on each slide.

#### Picrosirius red staining.

Slides were removed from –80˚C, thawed to room temperature (RT), and dried for 30 minutes before fixing in 4% paraformaldehyde in PBS for 15 minutes at RT. Following fixation, slides were washed 3 times in PBS for 5 minutes each, 2 times in deionized water for 5 minutes each, allowed to dry for 10 minutes at RT, and incubated in 0.5g Direct Red 80 solubilized in 500 mL Picric Acid for 1 hour at RT. Next, slides were washed twice with acidified water (2.5 mL glacial acetic acid in 500 mL deionized water) for 5 minutes each followed by 2 washes in deionized water for 5 minutes each. Tissue samples were then dehydrated in a series of ethanol washes (50%, 70%, 70%, 90%, 100%, 100%), incubated in xylenes twice for 5 minutes each, and mounted with Permount. Bright-field images were taken using a motorized Olympus IX83 microscope at 10***×*** magnification and stitched using the Olympus CellSense software to obtain an image of the complete tissue section. Images were converted to RGB stack and converted to grayscale. To calculate collagen fraction, the green channel was automatically thresholded and measured using ImageJ (NIH); it was then divided by the surface area of the tissue section based on thresholding the red channel to include only the tissue. Two or 3 sections per tissue were stained and quantified, and they were then averaged to get the percentage of collagen for each tissue.

#### Immunofluorescence staining.

Immunofluorescence staining was performed as previously reported (50). For Red FLuorescent Protein (RFP) and immune stains, slides were removed from –80˚C, thawed to RT for 30 minutes, and then fixed in ice-cold 100% acetone at –20˚C for 10 minutes. Following fixation, slides were air-dried for 10 minutes at RT, tissue sections were circled with Hydrophobic Barrier PAP Pen and allowed to dry at RT. Tissue sections were the rehydrated in PBS for 5 minutes at RT and were then blocked for 1 hour at RT using 10% normal goat serum (NGS) in PBS. After blocking, slides were incubated overnight at 4°C in a solution containing primary antibodies (rabbit anti–laminin 1+2 [1:500 dilution], rat anti–laminin 2α [1:1,000], rat anti-CD68 [1:50], and rabbit anti-RFP [1:100]) diluted in 10% NGS. Primary antibodies were then washed 3 times for 5 minutes with PBS at RT. Secondaries (1:500 dilution) and Hoescht 33342 (1:500 dilution) were added in PBS and incubated for 1 hour at RT in the dark. After incubation, slides were washed 3 times with PBS and a coverslip was mounted using Prolong Diamond florescence mounting medium. For GFP stain, slides were warmed to RT, and tissue sections were circled in PAP Pen, rehydrated in PBS 3 times for 5 minutes, blocked for 30 minutes at RT in mouse on mouse (MOM) blocking reagent, and incubated overnight at 4°C in primary antibodies (1:1,000 GFP and 1:500 anti–laminin 1+2) diluted in MOM Protein Concentrate. Following primary incubation, slides were washed 3 times for 5 minutes with PBS and incubated in secondary antibodies (AF488 anti-chicken, 1:250; AF555 anti-rabbit, 1:500, 1.5 μL/mL, Hoescht 33342) (Supplemental Table 3) diluted in MOM protein concentrate for 1 hour at RT. Finally, slides were washed 3 times for 5 minutes in PBS and were then mounted with Prolong Diamond. Slides were allowed to dry overnight and were then stored at 4°C until imaging. Images were acquired with a Nikon A1 confocal microscope equipped with Colibri 7 solid state light source and pseudo colored using ImageJ (NIH). They were then quantified using MATLAB to calculate the area of each region (defect, transition, intact, or the entire tissue section) that stained positive for CD68, GFP, or RFP as a percentage of the total area of that region. All images were acquired with the same laser intensity, exposure, and gain settings, and the threshold for what was considered positively stained was the same for all calculations.

#### Flow cytometry.

Mouse TAs were extracted by blunt dissection with sterile surgical tools, separately weighed, and minced into approximately 1 mm^2^ chunks using surgical scissors. Minced tissues were added to tubes containing Collagenase type II (0.2%) and Dispase II (2.5 U/mL) in 10 mL of DMEM. They were then placed in a 37°C bead bath for 1 hour. Samples were agitated every 5 minutes and pipette mixed every 30 minutes. The enzymes were then inactivated by the addition of 20% heat-inactivated FBS (HI-FBS) in Ham’s F10 media. The digest solution was passed through a 70 μm cell strainer, centrifuged at 350rpm for 5 minutes at 4°C, resuspended in FACS buffer (PBS with 2% BSA, 2 mM EDTA, and 0.01% sodium azide), and plated on a 96-well round-bottom plate. Cells were centrifuged at 350*g* for 2.5 minutes at 4°C and resuspended in a primary antibody cocktail including CD45-APC, CD68-FITC, and TREM2-PE for 30 minutes on ice. Cells were then centrifuged at 350*g* for 2.5 minutes at 4°C and resuspended in staining buffer containing 7-AAD for 10 minutes on ice, centrifuged, washed in FACS buffer, centrifuged, and resuspended in staining buffer for flow cytometry analysis. Prior to acquisition, cells were filtered through 40 mm cell strainers. Single-color controls were made using UltraComp eBeads compensation beads stained according to manufacturer’s protocol. Samples, single color, unstained, and fluorescence minus one (FMO) controls were acquired within 30 minutes on a BioRad Ze5 cytometer. Data were analyzed using FlowJo (version 10) with manual compensation. Graphs were made using FlowJo and ggplot2.

### spGEX sequencing

#### Sample preparation and sequencing.

Tissues were simultaneously flash frozen and OCT embedded as described above. RNA quality for each tissue was assessed using the QIAshredder and RNeasy Mini kit for RNA extraction according to manufacturer’s protocols, followed by BioAnalyzer RNA Pico assay; tissues with RNA integrity values above 7 were used for spGEX profiling. Permeabilization times were determined for each time point using the 10X Genomics Tissue Optimization kit and corresponding protocol. Tissues were permeabilized for 18 minutes. spGEX profiling was performed using the 10X Visium platform according to manufacturer’s instructions and sequenced on a NovaSeq 6000. H&E staining prior to tissue permeabilization was performed according to manufacturer’s protocols for the 10X Visium Tissue Optimization and Spatial Gene Expression products.

#### Data processing and analysis.

Manual image alignment was performed using 10X Loupe Browser. 10X SpaceRanger v1.3.0 software’s mkfastq and count command were run with default parameters and aligned to the mm10-2020-A genome. Tissues were annotated into zones using Loupe Browser v6. HDF5 matrix files and zone annotations were imported into R (https://www.r-project.org/) using the Seurat v4 package (20). Count data were normalized using SCTransform, followed by PCA dimension reduction and clustering using default parameters and the first 30 dimensions. Data set integration was performed using Seurat’s PrepSCTIntegration, FindIntegrationAnchors, and IntegrateData functions with default parameters for SCT normalization. PCA reduction, neighborhood analysis, clustering, and UMAP reduction were performed following integration. GO Term analysis was performed on scaled, normalized data using FindAllMarkers followed by ClusterProfiler’s enrichGO function on upregulated genes using the default settings (log fold change threshold of 0.25, p_adjusted threshold of 0.01, and genes expressed in at least 10% of the spots in the region). Mitochondrial and ribosomal genes were removed prior to GO Term enrichment analysis. Spot annotation with cell types was performed using Seurat label transfer with a matched scRNA-Seq reference generated from 3 mm quadricep defects at 7 or 14 dpi (9). Ligand-receptor analysis was performed on the 7 dpi scRNA-Seq data set used for label transfer using CellChat (22). Gene modules were generated using Seurat’s AddModuleScore function for overlays. Differential gene expression comparing the defect and transition zones between treated and untreated tissues was performed using MAST (29). Plots were generated using dittoSeq (51), ggplot2, EnhancedVolcano, and Seurat. Data are publically available at GSE205707 (https://www.ncbi.nlm.nih.gov/geo/query/acc.cgi?acc=GSE205707).

### Statistics

Experiments were repeated at least twice. Data are shown as mean ± SEM. Statistical analyses were performed in R using 2-sample Student’s *t* test assuming normal distribution and equal variances or 1-way ANOVA with Tukey’s post hoc analysis. All statistical tests performed were 2-tailed. Outliers were determined using the Tukey’s fences method with k = 1.5 and removed from further analysis. *P* values less than 0.05 were considered statistically significant.

### Study approval

All procedures were approved by the University Committee on the Use and Care of Animals at UM and the IACUC in Ann Arbor, Michigan, USA (protocol no. PRO000010663). Procedures were in accordance with the U.S. NIH.

## Author contributions

JAL, ECW, and BDS performed the experiments. JAL, ECW, and BDS analyzed the data. JAL and CAA designed the experiments. EB contributed reagents. CAA supervised the work. JAL and CAA wrote the manuscript with additions from other authors.

## Supplementary Material

Supplemental data

Supplemental tables 1-2

Supplemental table 3

## Version 1 02/23/2023

In-Press Preview

## Version 2 04/10/2023

Electronic publication
