# Supplementary material for: Spatiotemporal mapping of immune and stem cell dysregulation after volumetric muscle loss
Source: JCI Insight. 2023 Apr 10;8(7):e162835. doi: 10.1172/jci.insight.162835 (PMC10132146; doi:10.1172/jci.insight.162835)
Supplement: Supplemental data [file jciinsight-8-162835-s193.pdf]

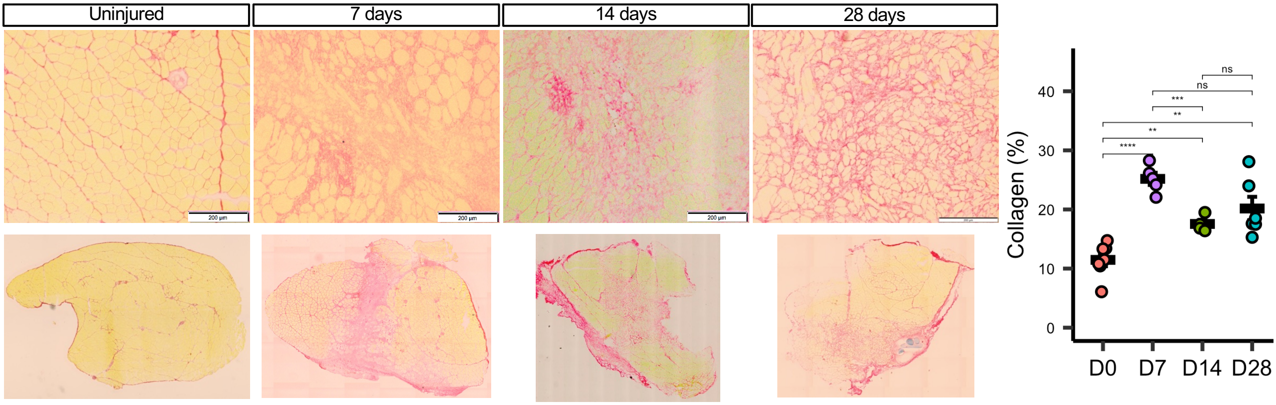

Supplemental Figure 1. Characterization of volumetric muscle loss in murine tibialis anterior muscle. Representative images of tissues stained with picrosirius red as well as quantification of collagen at each time point. n = 4-7 muscles. Full section 10X stitches were analyzed for each tissue. (Left) Representative stitched images used for analysis and insets showing detail of the injury area. Scale bar on insets indicates 200um. (Right) Quantification results. **p<0.01, ***p<0.001 ,****p<0.0001 by one-way ANOVA and Tukey's post-hoc analysis.

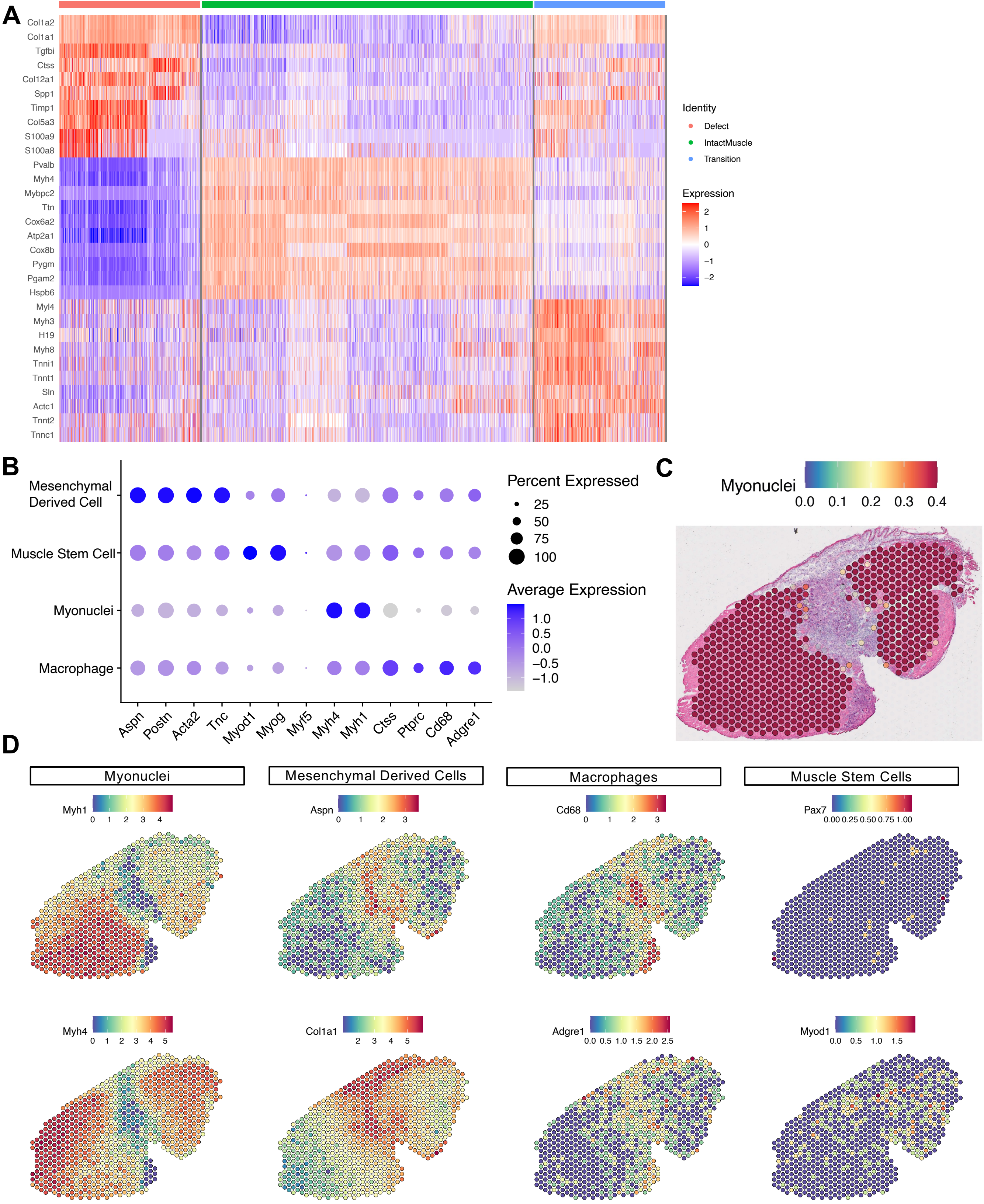

Supplemental Figure 2. Extended analysis of murine 7-dpi spGEX datasets. (A) Heatmap of gene signatures for each zone at 7-dpi. (B) Dot plot showing the expression of known cell-type marker genes in annotated spGEX spots. Color bar indicates scaled expression (z-score). (C) Spatial overlay of myonuclei prediction scores for each spot. Color bar indicates prediction score. (D) Overlays of selected cell marker genes. Color bar shows the SCTransform expression level.

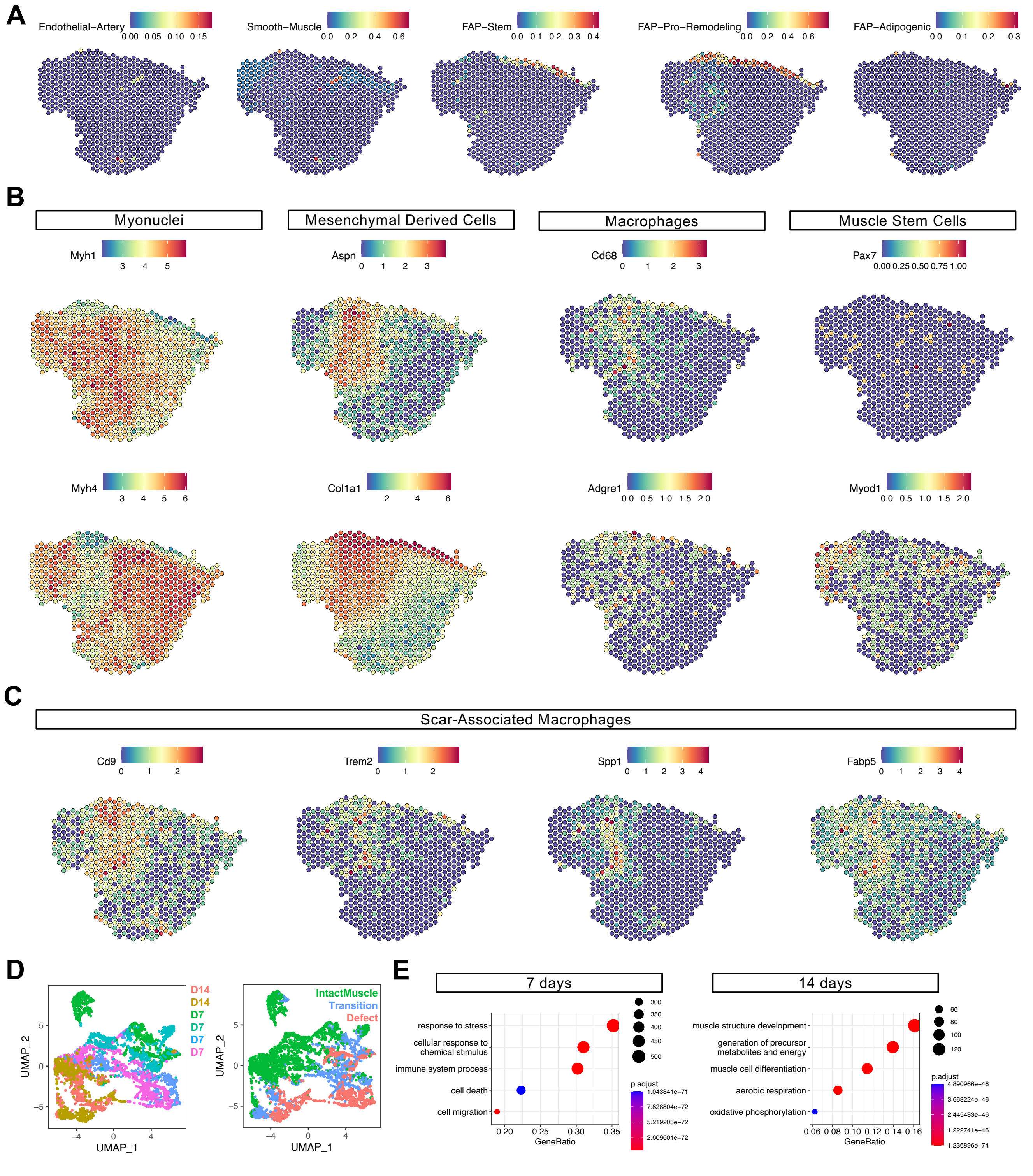

Supplemental Figure 3. Extended analysis of murine 14-dpi spGEX datasets. (A) Prediction scores for remaining cell types based on Seurat label transfer with timepoint matched scRNA-Seq dataset. Color bars show prediction scores. (B) Overlays of selected cell marker genes. Color bar shows the SCT-transformed expression level. (C) Overlays of marker genes for scar-associated macrophages. Color bar shows the SCTransformed expression level. (D) UMAP plots colored by sample and zone. (E) Upregulated GO-Terms within the transition and defect zone at 7-dpi compared to 14-dpi (left) and 14-dpi compared to 7-dpi (right).

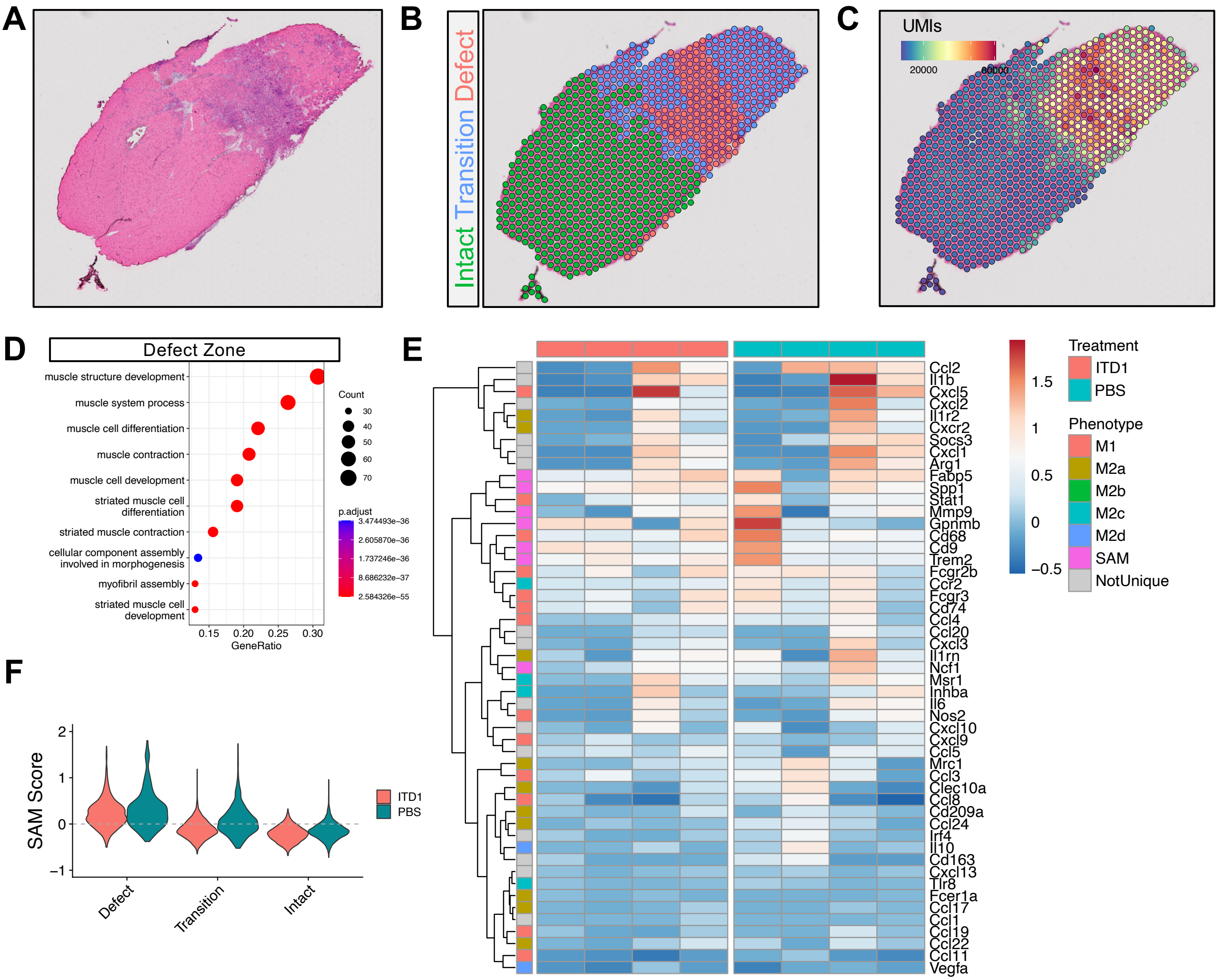

Supplemental Figure 4. Characterization of ITD1-treated TA VML defects and differences among the transcriptional landscape of the defect zone compared to vehicle controls. (A) Representative image of H&E-stained tissue treated with ITD1. (B) Representative tissue annotation into the three zones. (C) Distribution of unique molecular identifiers shows higher read counts at the location of the defect and transitional zones, consistent with untreated tissues. (D) GO Term analysis comparing ITD1 vs PBS-treated tissues in the defect zone. Differentially expressed genes were calculated using Wilcoxon Sum Rank Test with post hoc analysis. Log2 fold change > 0.25 and p_adjusted > 0.05 was considered significant. (E) Heatmap of the expression of genes associated with macrophage phenotypes according to treatment. Scale bar indicates z-score of average gene expression for each replicate in each treatment. n = 4 tissues from 2 male and 2 female mice per treatment. (F) Seurat module score for genes associated with scar-associated macrophages, comparing treatments and zones suggests reductions in SAMs as a result of ITD1 treatment. $p<0.05$ for all comparisons (across zones and treatments) by two-way ANOVA with Tukey's post-hoc analysis.
