# Supplementary material for: Spatiotemporal mapping of immune and stem cell dysregulation after volumetric muscle loss
Source: JCI Insight. 2023 Apr 10;8(7):e162835. doi: 10.1172/jci.insight.162835 (PMC10132146; doi:10.1172/jci.insight.162835)
Supplement: Supplemental table 3 [file jciinsight-8-162835-s195.pdf]

KEY RESOURCES TABLE

REAGENT or RESOURCE	SOURCE	IDENTIFIER
**Antibodies**		
Rat anti-mouse Ly6G	Abcam	ab25377; RRID:AB_470492
Rat anti-mouse CD68	BioRad	MCA1957; RRID:AB_322219
Rabbit anti-mouse RFP	Rockland	600-401-379; RRID:AB_2209751
Chicken anti-mouse GFP	Abcam	ab13970; RRID:AB300798
Rabbit anti-mouse Laminin 1+2	Abcam	ab7463; RRID:AB_305933
APC anti-mouse CD45	BioLegend	103112; RRID:AB_312977
FITC anti-mouse CD68	BioLegend	137006; RRID:AB_10578412
PE anti-mouse/anti-human TREM2	R&D Systems	FAB17291P; RRID:AB_884528
Goat anti-chicken IgY (H+L), Alexa Fluor 488 conjugate	Thermo Fisher	A32931TR; RRID:AB_2866499
Goat anti-rat (H+L), Alexa Fluor 647 conjugate	Thermo Fisher	A21247; RRID:AB_141778
Goat anti-rabbit (H+L), Alexa Fluor 555 conjugate	Thermo Fisher	A21247; RRID:AB_141778
**Chemicals, Peptides, and Recombinant Proteins**		
Dispase II (activity ≥0.5 units/mg solid)	Sigma	D4693-1G
Collagenase Type II (654 U/mg, non-specific proteolytic activity 487 U/mg)	Life Technologies	17101015
DMEM, high glucose, pyruvate	Life Technologies	11995065
Ham's F-10 Nutrient Mix	Life Technologies	11550043
Fetal Bovine Serum	Life Technologies	10437028
Normal Goat Serum	Abcam	Ab7481; RRID:AB_2716553
Mouse on Mouse blocking reagent	Vector Labs	MKB-2213
Tissue Plus O.C.T Compound	Fisher Scientific	23-730-571
Hematoxylin	Ricca Chemical Company	3530-16
Eosin	EMD-Millipore	588X-75
Magnesium Sulfate Heptahydrate	Sigma Aldrich	63138-250G
Sodium Bicarbonate	Sigma Aldrich	S5761
SafeClear II	Fisher Scientific	23-044192
Direct Red 80	Fisher Scientific	AAB2169306
Picric Acid	Sigma Aldrich	P6744-1GA
Glacial Acetic Acid	Sigma Aldrich	BP2401-500
Xylenes	Sigma Aldrich	534056-4L
Permount	Fisher Scientific	SP15-100
Bovine Serum Albumin	Fisher Scientific	BP9703-100
Prolong Diamond	Thermo Fisher	P36965
Tween-20	Sigma Aldrich	P1379
TritonX-100	Sigma Aldrich	T8787
Hoechst 33342	Thermo Fisher	H3570
7-AAD	BioLegend	420403
**Critical Commercial Assays**		
QIAshredder	Qiagen	79656
Qiagen RNeasy Mini Kit	Qiagen	217084
BioAnalyzer RNA Pico Assay	Agilent	5067-1514
Tissue Optimization	10x Genomics	1000193
Visium Spatial Gene Expression	10x Genomics	1000187

**Deposited Data**		
spGEX datasets	This Manuscript	GSE205707
**Experimental Models: Organisms/Strains**		
C57BL/6J wild-type mice (3 months)	Jackson Labs	Strain 000664; RRID:IMSR_JAX:000664
PDGFRa^{EGFP} mice (3 months)	University of Michigan	Strain 007669; RRID:IMSR_JAX:007669
Pax7Cre$^{ER/+}$;Rosa26$^{dTomato/+}$ mice (3 months)	University of Michigan	Strain 017763 crossed with strain 007676
**Software and Algorithms**		
SpaceRanger v1.3.0	10x Genomics	https://support.10xgenomics.com/spatial-gene-expression/software/downloads
R v4.1.2	The R Foundation for Statistical Computing	https://www.r-project.org/ RRID:SCR_001905
MATLAB_R2020b	MathWorks	https://www.mathworks.com/products/matlab.html
Seurat v4.1.0	Butler et al. 2019	https://satijalab.org/seurat/ RRID:SCR_007322
CellChat v1.0.0	Jin 2021	https://github.com/sqjin/CellChat
ggplot2 v3.3.5	Wickham 2016	https://ggplot2.tidyverse.org RRID:SCR_014601
dplyr v1.0.8	Wickham 2016	https://dplyr.tidyverse.org/ RRID:SCR_016708
Tidyverse v1.3.0		https://www.tidyverse.org/ RRID:SCR_019186
ImageJ v2.1.0		https://imagej.net/ImageJ RRID:SCR_002285
MAST v1.20.0	Finak 2015	https://github.com/RGLab/MAST RRID:SCR_016340
EnhancedVolcano v.1.12.0	Blighe K. et al. 2019	https://github.com/kevinblighe/EnhancedVolcano
QuPath	Bankhead et al. 2017	RRID:SCR_018257
dittoSeq	Bunis et al. 2020	
clusterProfiler	Wu et al. 2021	RRID:SCR_016884
AnnotationDbi	Pagés et al. 2022	
**Other**		
Bioinformatics analysis code	This manuscript	https://github.com/larouchej/Spatial_VML
